# Effect of Maternal Steroid on Developing Diaphragm Integrity

**DOI:** 10.1371/journal.pone.0093224

**Published:** 2014-03-28

**Authors:** Yong Song, Denise L. Demmer, Gavin J. Pinniger, Tina Lavin, Mia V. MacMillan, Jane J. Pillow, Anthony J. Bakker

**Affiliations:** 1 School of Anatomy, Physiology and Human Biology, The University of Western Australia, Perth, Western Australia, Australia; 2 Centre for Neonatal Research and Education, School of Paediatrics and Child Health, The University of Western Australia, Perth, Western Australia, Australia; 3 Women and Newborns Health Service, c/-King Edward Memorial and Princess Margaret Hospitals, Perth, Western Australia, Australia; Hôpital Robert Debré, France

## Abstract

Antenatal steroids reduce the severity of initial respiratory distress of premature newborn babies but may have an adverse impact on other body organs. The study aimed to examine the effect of maternal steroids on postnatal respiratory muscle function during development and elucidate the mechanisms underlying the potential myopathy in newborn rats. Pregnant rats were treated with intramuscular injections of 0.5 mg/kg betamethasone 7 d and 3 d before birth. Newborn diaphragms were dissected for assessment of contractile function at 2 d, 7 d or 21 d postnatal age (PNA), compared with age-matched controls. The expression of myosin heavy chain (MHC) isoforms and atrophy-related genes and activity of intracellular molecular signalling were measured using quantitative PCR and/or Western blot. With advancing PNA, neonatal MHC gene expression decreased progressively while MHC IIb and IIx isoforms increased. Protein metabolic signalling showed high baseline activity at 2 d PNA, and significantly declined at 7 d and 21 d. Antenatal administration of betamethasone significantly decreased diaphragm force production, fatigue resistance, total fast fibre content and anabolic signalling activity (Akt and 4E-BP1) in 21 d diaphragm. These responses were not observed in 2 d or 7 d postnatal diaphragm. Results demonstrate that maternal betamethasone treatment causes postnatal diaphragmatic dysfunction at 21 d PNA, which is attributed to MHC II protein loss and impairment of the anabolic signalling pathway. Developmental modifications in MHC fibre composition and protein signalling account for the age-specific diaphragm dysfunction.

## Introduction

Women at risk of preterm birth are routinely given two 12 mg doses of betamethasone (a glucocorticoid: a class of steroid hormones) at 24 h intervals, to promote structural and functional maturation of the fetal lung. Infants born preterm are therefore frequently exposed to antenatal glucocorticoid treatment.

Preterm infants have reduced diaphragm contractility compared to their term gestation counterparts [1], [2]. As preterm infants often breathe against an increased mechanical load, diaphragm integrity is particularly critical for self-sufficient ventilation. In adults, there is considerable evidence implicating steroid administration in the development of significant respiratory muscle weakness [3]–[6]. Antenatal steroid exposure could precipitate or accelerate the development of postnatal respiratory failure through inducing additional compromise of the functional and phenotypic integrity of the fragile immature diaphragm. The contribution of antenatal steroid exposure to preterm diaphragm dysfunction and increased susceptibility to respiratory failure is unknown.

Adult animal studies show that diaphragmatic structure and function is severely compromised by the use of systemic steroids [3]–[6], characterised by diaphragmatic atrophy and weakness. Glucocorticoid treatment reduces maximal isometric tetanic tension (normalized to muscle cross-sectional area) in adult rat diaphragms [4], accompanied by a selective atrophy of type IIx and IIb fibres [3], [6]–[8]. Glucocorticoid-induced skeletal muscle atrophy is associated with reduced protein synthesis and accelerated protein breakdown [9]. The inhibitory effect of glucocorticoids on protein synthesis is exerted via down-regulation of insulin-like growth factor-1 (IGF-1) signalling and de-activation of two key translation initiation factors (p70S6K and 4E-BP1) [9]. The stimulatory effect of glucocorticoid on muscle proteolysis occurs via activation of a major cellular proteolytic system (ubiquitin proteasome pathway, UPP) [9].

Diaphragmatic impairment is primarily described in reference to the adult subject and late postnatal period [4], [10], [11]. However, the premature diaphragm is weaker and more susceptible to injury compared with that at term owing to differences in fibre-type composition [2], [12] and oxidative capacity [13]. Since prenatal organ development is largely under the control of genetic programming [14], environmental insults encountered *in utero* may alter gene expression, adversely affect the metabolic and endocrine balance of affected individuals and subsequently lead to dysfunction later in life [14]. In addition, as steroid actions depend on the developmental status of the diaphragm, time of steroid administration during muscular development is regarded as a major determinant of features of the steroid myopathy [15]. Thus *in utero* steroid effects on the developing muscles (particularly premature) are likely to differ greatly from those observed on the mature muscles and be influenced by maturational change.

The aim of the present study was to evaluate functional and phenotypic changes in the developing diaphragm following maternal steroid exposure and to explore the mechanisms underlying any potential myofibre dysfunction. We hypothesised that antenatal exposure to steroids would promote structural and physiological changes in the offspring diaphragm resulting in postnatal respiratory muscle weakness. To test this hypothesis, we used a newborn rat model to analyse diaphragm contractile properties, fibre type composition, protein synthesis pathway activity and expression of atrophy related genes at different postnatal ages (PNA) (2 d, 7 d and 21 d) in response to antenatal steroid exposure. Like preterm infants, newborn rats have a high proportion of neonatal and fast type II fibres [16], which are more susceptible to injurious factors than type I fibres [17], [18]. The respiratory muscle fibre type transition that occurs during late gestation (24–42 w) in the human fetus and early postnatal (0–21 d) rat are similar. Therefore, the term newborn rat is an appropriate model for studying development of the immature diaphragm [19].

## Materials and Methods

### Animals and Experimental Design

All experiments were approved by the Animal Ethics Committee of the University of Western Australia (Permit Number: RA/3/100/1097). Pregnant Wistar rats were housed in separate cages during gestation and they were fed ad libitum. A 12 h light-dark cycle was provided. Room temperature was maintained at 25°C. The rats were randomly assigned either to the betamethasone treated group (given Celestone from Shering Plough intramuscularly with 0.5 mg/kg at 15 d and 19 d gestational age) or the untreated control group. After spontaneous delivery at term (22 d), newborn rats were grown to 2 d, 7 d and 21 d from each litter. Euthanasia was induced by an overdose of pentobarbitone (160 mg/kg) through intraperitoneal injection. After terminal anesthesia the pup diaphragm strips were immediately examined for contractile function, and tissue samples from different pups were frozen in liquid nitrogen for analysis of MHC composition and protein signalling.

### Muscle Contractile Properties

Dissection and preparation of the diaphragm strips and the measurement of diaphragm contractile properties were described previously [2], [20]. The diaphragm was rapidly removed from the animal and maintained in a petri dish containing Krebs physiological salt solution bubbled with 95% O_2_/5% CO_2_ (pH 7.4) at room temperature (∼21–23°C). Longitudinal diaphragm strips with an intact section of the central tendon at one end, and a section of attached rib at the other, were isolated for experimentation. The strips were mounted onto an *in vitro* muscle test system (model 1205, Aurora Scientific In., Aurora, Canada) using surgical thread, and maintained in Krebs physiological salt solution maintained at 25°C and continuously bubbled with 95% O_2_/5% CO_2_ (pH 7.4).

All force measurements were recorded at optimal length (L_o_) which is defined as the muscle fibre length at which maximal twitch force was generated. All contractions were elicited using a supramaximal square wave stimulus of 0.2 ms duration. Single twitch contractions were analysed for peak twitch force (N/cm^2^), time to peak twitch tension (TTP) and half relaxation time (½ RT). The force frequency relationship was then determined from tetanic force responses elicited for 700 ms at stimulation frequencies of 5, 10, 20, 40, 60, 80, 100 and 120 Hz. Susceptibility to fatigue was evaluated by stimulating once every 5 s for 700 ms at 80 Hz for a total of 240 s.

At the completion of the contractile measurements, optimal fibre length (mm) was measured using electronic calipers, and the wet muscle mass (mg) determined for estimation of muscle cross sectional area (CSA). CSA was estimated by dividing the muscle mass by the product of the optimal fibre length and muscle density (1.056 g/cm^3^) [4], and the values obtained were used for the calculation of muscle specific force. Muscle specific force is force normalised to muscle cross sectional area (N/cm^2^). It provides a measure of force output that is independent of muscle size, and can be used to compare contractility in muscle preparations of different ages or proportions. Therefore, maximal specific force data cannot be used as an indicator of atrophy in these experiments.

### RNA Isolation, Reverse Transcription and Quantitative PCR

RNA purification, reverse transcription and quantitative PCR were performed as described previously [2], [13]. The primers of MHC gene (*MHC neonatal*, *MHC I*, *MHC IIa*, *MHC IIb* and *MHC IIx*) and atrophic gene (*MAFbx* and *MuRF1*) were described previously by other investigators [21], [22]. The fluorescence signal was analyzed and normalized against GAPDH which showed stability amongst the different groups. Relative expression levels were obtained using the 2^−ΔΔCT^ method and presented as fold change relative to the control group of 2 d PNA.

### Muscle Protein Extraction and Western Blot Analysis

The whole cell lysate was prepared as described previously [13]. Protein concentration was measured using the Bradford method (Sigma, Sydney, Australia). 20 μg protein samples were resolved on 4–15% TGX Stain-Free gels (Bio-Rad, Gladesville, NSW, Australia) and protein was transferred onto Nitrocellulose membrane using a Trans Turbo Blot system (Bio-Rad). Stain-Free technology enables fluorescent visualization of 1-D SDS-PAGE gels and corresponding blots using Bio-Rad ChemiDoc MP imaging system. Total protein was visualized on the UV setting in the imaging system and calculated on both the SDS-PAGE gel and the membrane to ensure the quality of SDS-PAGE separations and the transfer efficiency of the blotting process. Western blotting was performed with primary antibodies for phosphorylated (p)-Akt (Ser473) (9271), total Akt (9272), p-4E-BP1 (Thr70) (9455), total 4E-BP1 (9452) from Cell Signalling Technology (Carlsbad, CA, USA), and MHC I (NCL-MHCs) and MHC II (NCL-MHCf) from Novocastra (Newcastle, UK). Bound antibodies were detected with either anti-rabbit or anti-mouse immunoglobulin conjugated with horseradish peroxidises (Cell Signalling Technology). After adding a chemiluminescent substrate (Thermo Scientific, Massachusetts, USA), immunoreactive protein signals were detected and quantified using ChemiDoc MP Imaging System. The values for each protein were normalized to total protein content of the same lane on the blot.

### Data Analysis

Sigmaplot (version 12.5, Systat Software Inc, San Jose, USA) was used for statistical analysis. Data were assessed for normal distribution and non-normally distributed data were log_10_-transformed. Differences among multiple groups were assessed using two-way ANOVA with PNA and group assignment as the two factors. If no interaction between PNA and treatment was found, post hoc tests of least significant differences were used for direct mean comparisons. Where an interaction between factors (age and treatment) was found, differences within postnatal control groups were determined with a 1-way ANOVA followed by post hoc least significant difference tests and differences between steroid and control groups were determined by 2-tail *t* tests. Differences were accepted as significant at *p*<0.05. Data were presented as mean (SE) or median (range) unless specified otherwise.

## Results

### Animal Characteristics

Betamethasone (0.5 mg/kg) was given antenatally in pregnant rats at 3 d and 7 d prior to expected birth (no preterm labour). The dose and timing of steroid administrated did not cause a significant difference in body weight or diaphragm weight between steroid treatment groups and age-matched control groups (Table 1). As expected, there was a significant effect of age on body weight and diaphragm weight over the course of postnatal development.

**Table 1:** Body and diaphragm weights.

	2 d		7 d		21 d	
	Con	Beta	Con	Beta	Con	Beta
n	4	8	8	4	5	6
Body weight (g)	7.0±0.7	6.7±1.0	16.2±1.6*	17.6±0.7	60.4± 24* ^ ∧^	60.1±7.4
Dia weight (mg)	28.3±5.3	26.1±5.8	62.9±9.2*	66.0±2.7	245.9±69.4* ^ ∧^	202.8±26.3
Ratio (%)	0.42±0.12	0.40±0.09	0.39±0.04	0.38±0.03	0.40±0.10	0.34±0.03

Con: control; Beta: betamethasone; Dia: diaphragm; Ratio: diaphragm weight/body weight. Values are mean ± SD.

*^ ∧^indicates *p*<0.001 compared with controls of 2 d and 7 d respectively.

### Contractile Measurements

**Figure 1:**
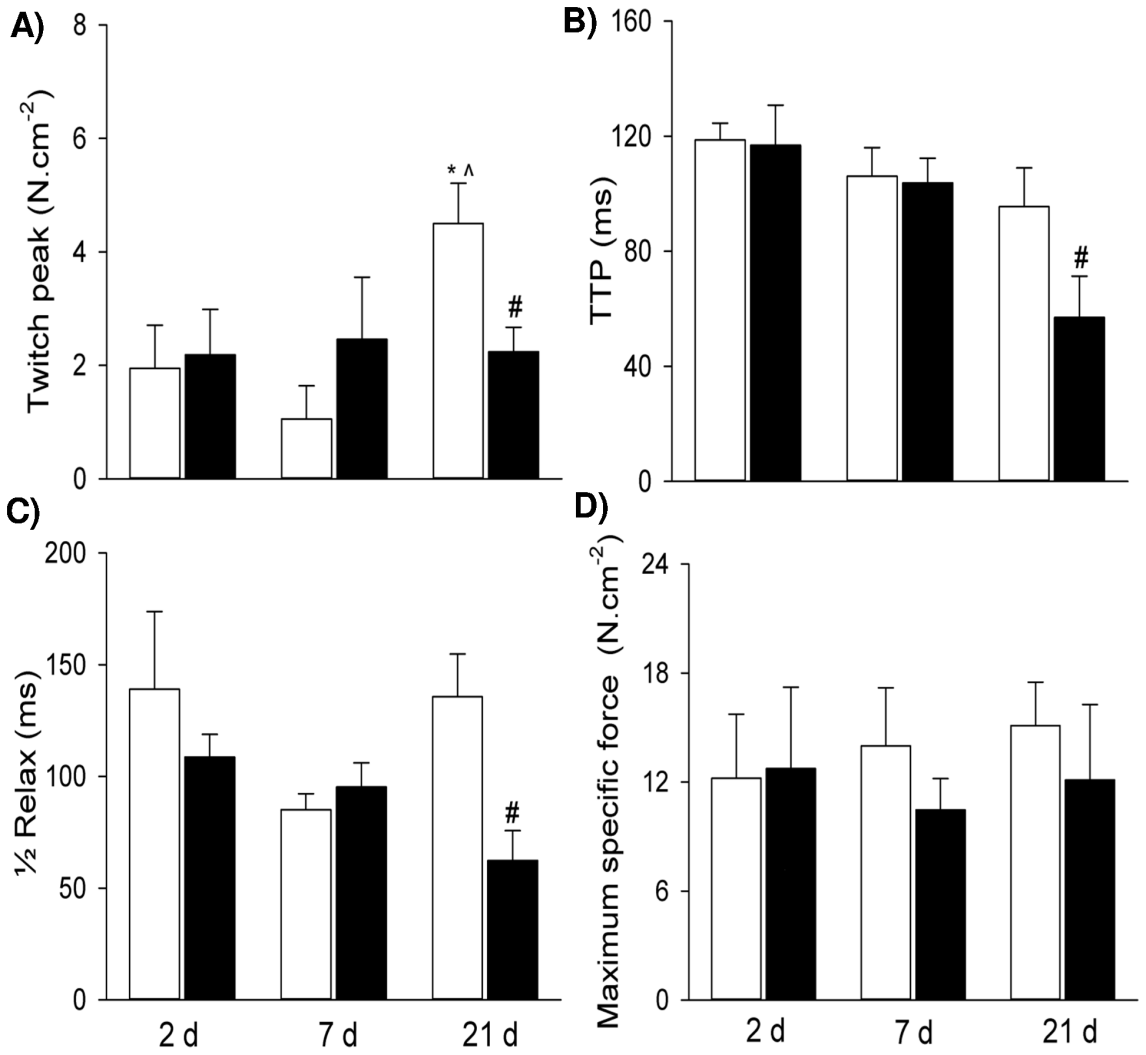
Fetal Diaphragm Contractile Properties. Twitch peak force (A), time to peak (B), half relaxation time (C) and maximum specific force (D) in the controls and maternal steroid treatment groups of 2 d, 7 d and 21 d postnatal age (n = 6 for each group). Values are Mean (SE). White and black bars refer to the control and steroid treatment groups respectively. ^* ∧ #^ indicates *p*<0.05 compared with controls of 2 d, 7 d and 21 d respectively. TTP: time to peak; ½ Relax: half relaxation time.

Postnatal diaphragm peak twitch force increased dramatically at 21 d, compared with 2 d and 7 d (Figure 1A). The antenatal administration of betamethasone significantly decreased the mean peak twitch force of pup diaphragm preparations at 21 d PNA by approximately 50%, compared to the corresponding control groups (*p*<0.05). However, no effect of betamethasone exposure on peak twitch force was evident at the 2 d and 7 d time points (Figure 1A). Betamethasone exposure also significantly reduced the contraction (TTP) and relaxation (½ RT) times of the twitch responses in diaphragm preparations from 21 d pups, to approximately 40% and 50% of controls, respectively (*p*<0.05). However, betamethasone had no effect on contraction and relaxation times in the 2 d and 7 d diaphragm groups compared to controls (*p*>0.05) (Figure 1B and 1C). The decreased TTP and ½ RT times of the twitch responses in the 21 d betamethasone-exposed diaphragm strips may indicate changes in sarcoplasmic reticulum (SR) Ca^2+^ handling, or simply reflect the decreased twitch peak force found in these responses. Therefore, we compared the twitch TTP/peak and ½ RT/peak ratios (to normalise these values for peak force). No significant differences in either the mean TTP/peak ratio or the mean ½ RT/peak ratio were found between the two groups.

Betamethasone exposure had no significant effect on maximum specific force (N/cm^2^) at any postnatal time point (Figure 1D). Maximum specific force levels elicited under similar conditions in control adult rat diaphragm strips were significantly higher than those from control neonatal diaphragm (19.61±0.66 N/cm^2^, *p*<0.05).

**Figure 2:**
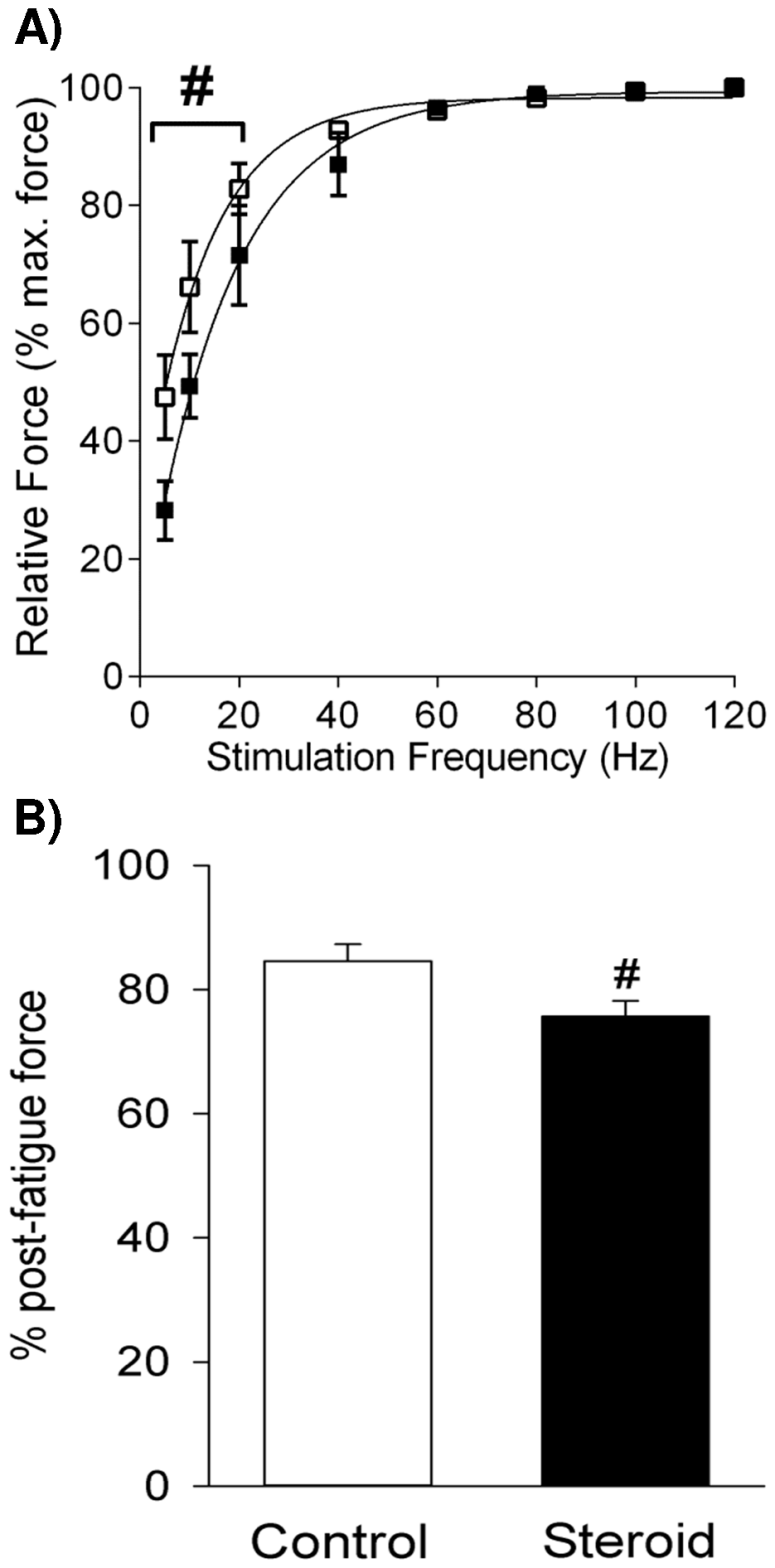
Force-Frequency Relationship and Post-Fatigue Force in 21 d Diaphragm. Force-stimulation frequency relationship (A) and post-fatigue force (B) in the 21 d betamethasone and control groups (n = 6 for each group). White and black box plots refer to the control and steroid treatment groups respectively. Values are Mean (SE). ^#^ indicates *p*<0.05 compared with the control.

As betamethasone decreased submaximal (twitch) force but not maximal force production in the 21 d group, we examined the effect of betamethasone administration on submaximal force production by comparing the force-stimulation frequency relationship in the 21 d betamethasone and control groups. Figure 2A shows that betamethasone exposure shifted the force-stimulation frequency relationship to the right in response to betamethasone exposure. Relative force in the betamethasone group was significantly reduced to 60%, 75% and 86% of that observed in controls at stimulation frequencies of 5 Hz, 10 Hz and 20 Hz, respectively.

The effect of betamethasone exposure on the susceptibility to fatigue was assessed by exposing the diaphragm preparations to 4 min of fatiguing stimulation. No significant differences in fatigability were observed between betamethasone treated and control preparations in the 2 d and 7 d groups (data not shown). However, in the 21 d group the post-fatigue force value in the betamethasone exposed group was of 75.7% of initial force, which was significantly lower than the control post-fatigue value of 84.6% of initial force (*p*<0.05) (Figure 2B).

### MHC Gene and Protein Expression

**Figure 3:**
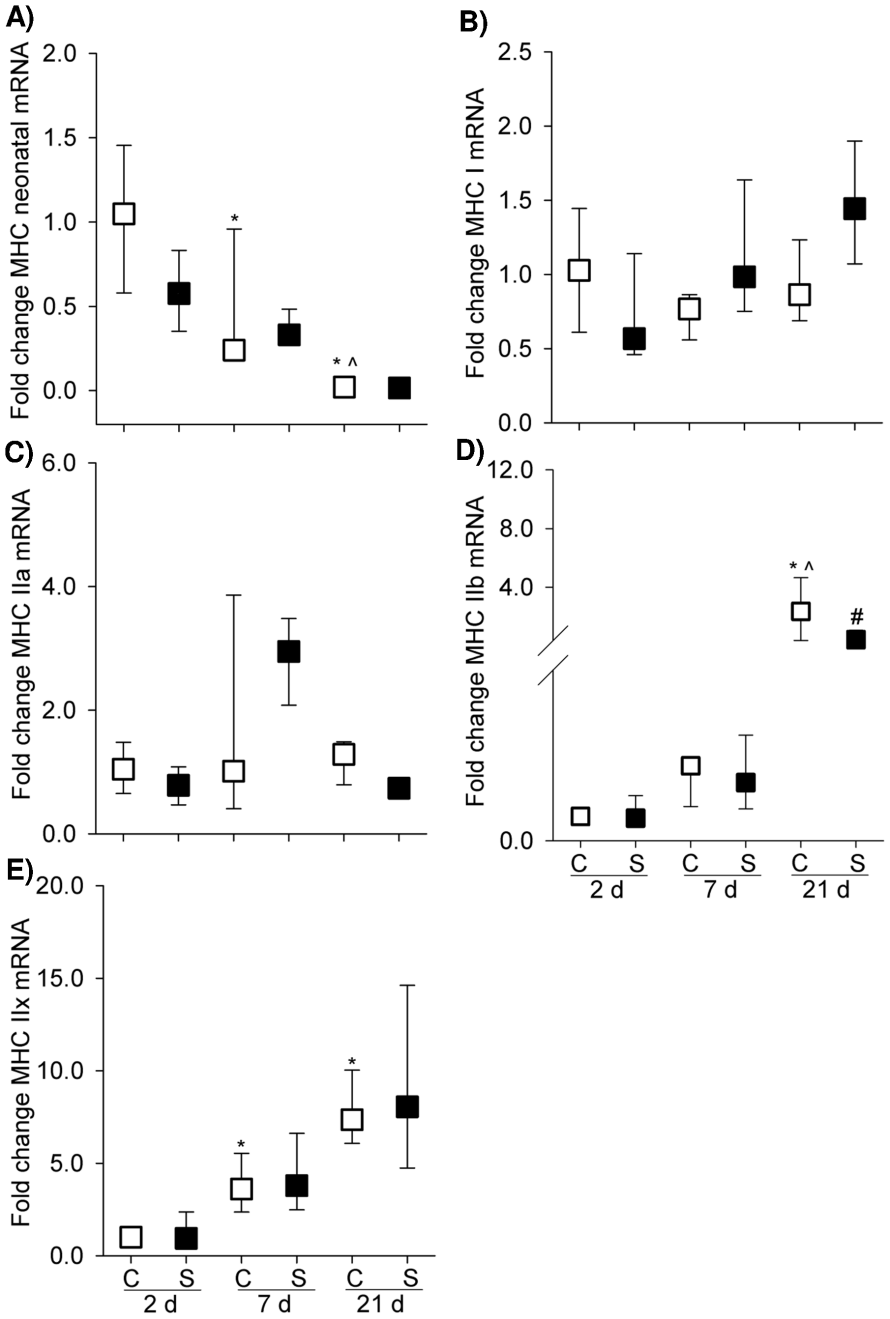
MHC Gene Expression. Graphs show MHC neonatal (A), MHC I (B), MHC IIa (C), MHC IIb (D) and MHC IIx (E) mRNA level in the controls and maternal steroid treatment groups of 2 d (n = 4 for control, n = 8 for steroid group), 7 d (n = 7 for control, n = 4 for steroid group) and 21 d (n = 5 for both control and steroid groups) postnatal age. Values are Median (25^th^, 75^th^ centile), expressed as fold change relative to 2 d control group. White and black box plots refer to the control and steroid treatment groups respectively. ^* ∧ #^ indicates *p*<0.05 compared with controls of 2 d, 7 d and 21 d respectively. MHC: myosin heavy chain. C: control; S: steroid.

Within postnatal development from 2 d to 21 d, there was a progressive decrease of MHC neonatal isoform mRNA transcripts in the naïve pup diaphragm (*p*<0.001, Figure 3A). MHC neonatal gene expression was down-regulated in 7 d and 21 d PNA by approximately 2.2 and 49.9 fold relative to 2 d PNA (*p*<0.001), respectively. Compared with 7 d PNA, 21 d postnatal pup diaphragm demonstrated a further decrease in MHC neonatal mRNA level (*p*<0.05). However, postnatal development did not significantly change the amount of MHC I mRNA (Figure 3B). Further, three MHC II isoforms were examined for developmental change with different patterns observed between MHC IIa and MHC IIb/IIx. There was no significant difference in MHC IIa transcripts across the different groups (Figure 3C), whereas an increasing trend in MHC IIb and MHC IIx transcripts: mRNA expression peaked at 21 d with a 319 and 7.9 fold increase from 2 d to 21 d, respectively (Figure 3D and 3E).

MHC IIb gene expression was significantly down-regulated at 21 d PNA following maternal steroid treatment, compared with the corresponding control group (*p*<0.05) (Figure 3D). No difference in MHC IIb was observed at other postnatal times or in other MHC isoforms.

**Figure 4:**
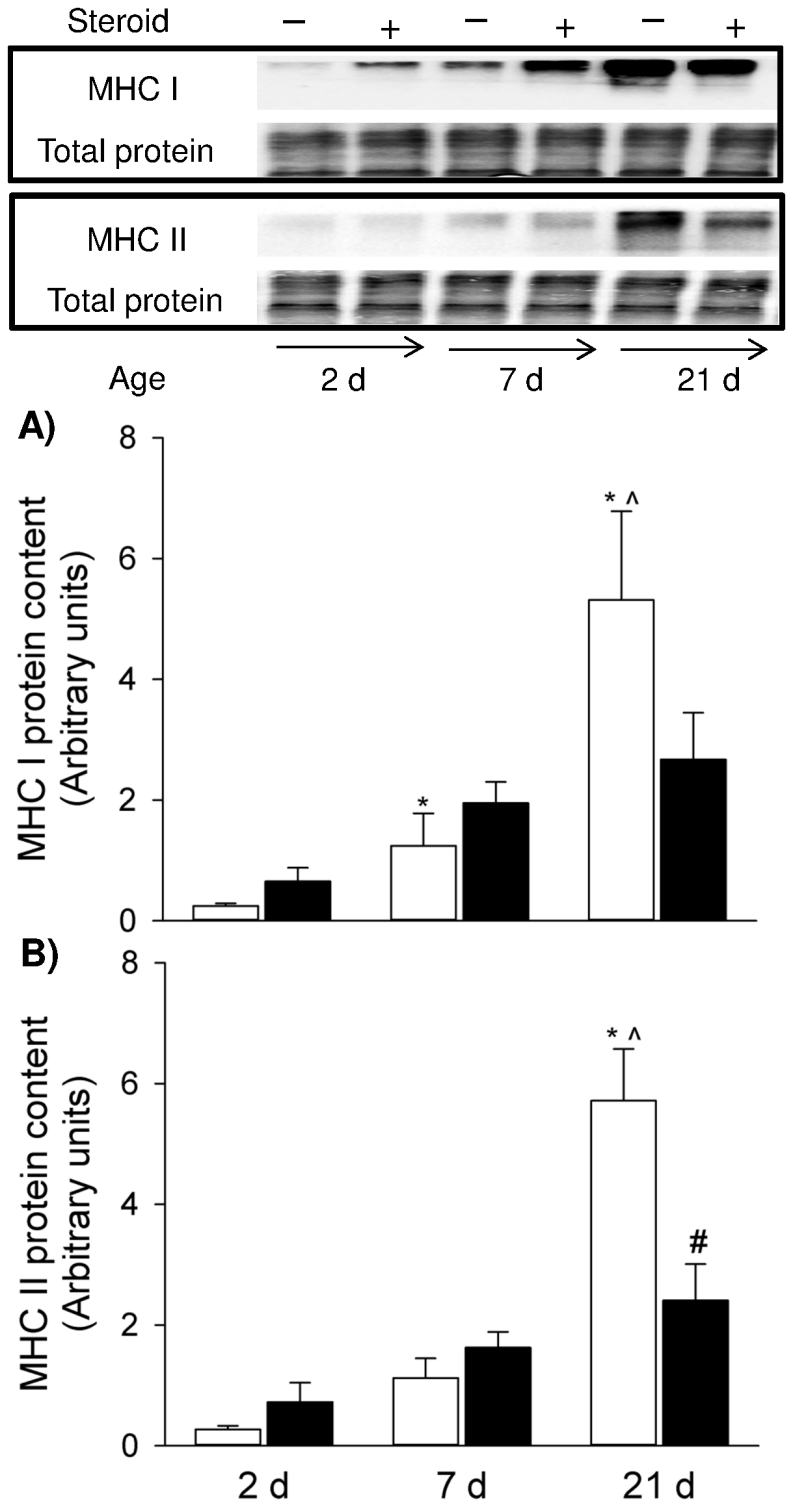
MHC Protein Expression. Western blots illustrate expression of MHC I and II proteins using representative samples above the graphs. Graphs show MHC I (A) and MHC II (B) protein content in the controls and maternal steroid treatment groups of 2 d (n = 4 for both control and steroid groups), 7 d (n = 4 for both control and steroid groups) and 21 d (n = 5 for both control and steroid groups) postnatal age. Values are Mean (SE). White and black bars refer to the control and steroid treatment groups respectively. ^* ∧ #^ indicates *p*<0.05 compared with controls of 2 d, 7 d and 21 d respectively. MHC: myosin heavy chain.

We further examined changes in MHC I and II protein expression in response to postnatal maturation and maternal steroid in the pup diaphragm. There was a significant correlation between postnatal development and protein content of MHC I (r^2^ = 0.806, *p*<0.0001) and MHC II (r^2^ = 0.875, *p*<0.0001). Both MHC I and II protein increased with increasing maturation (Figure 4A and 4B): compared to the 2 d PNA newborn diaphragm, MHC I and II proteins were 21.8 and 21.3 fold higher at 21 d PNA. In accordance with MHC IIb gene expression data, total MHC II protein was significantly reduced after maternal steroid treatment whilst MHC I protein content was not significantly different after steroid exposure (*p* = 0.150).

### Molecular Signalling

To identify signal transduction cascades involved in maternal steroid induced diaphragm dysfunction, we evaluated two key intracellular mediators of anabolic signalling (Akt and 4E-BP1). Normally, the activity of Akt and 4E-BP1 are presented as the ratio of the phosphorylated (active) forms over the total Akt and 4E-BP1 respectively. However, in the present study postnatal development also altered the total 4E-BP1 protein content. Therefore, we also measured phosphorylated forms of Akt and 4E-BP1/total protein ratio as activity of Akt and 4E-BP1 signalling.

**Figure 5:**
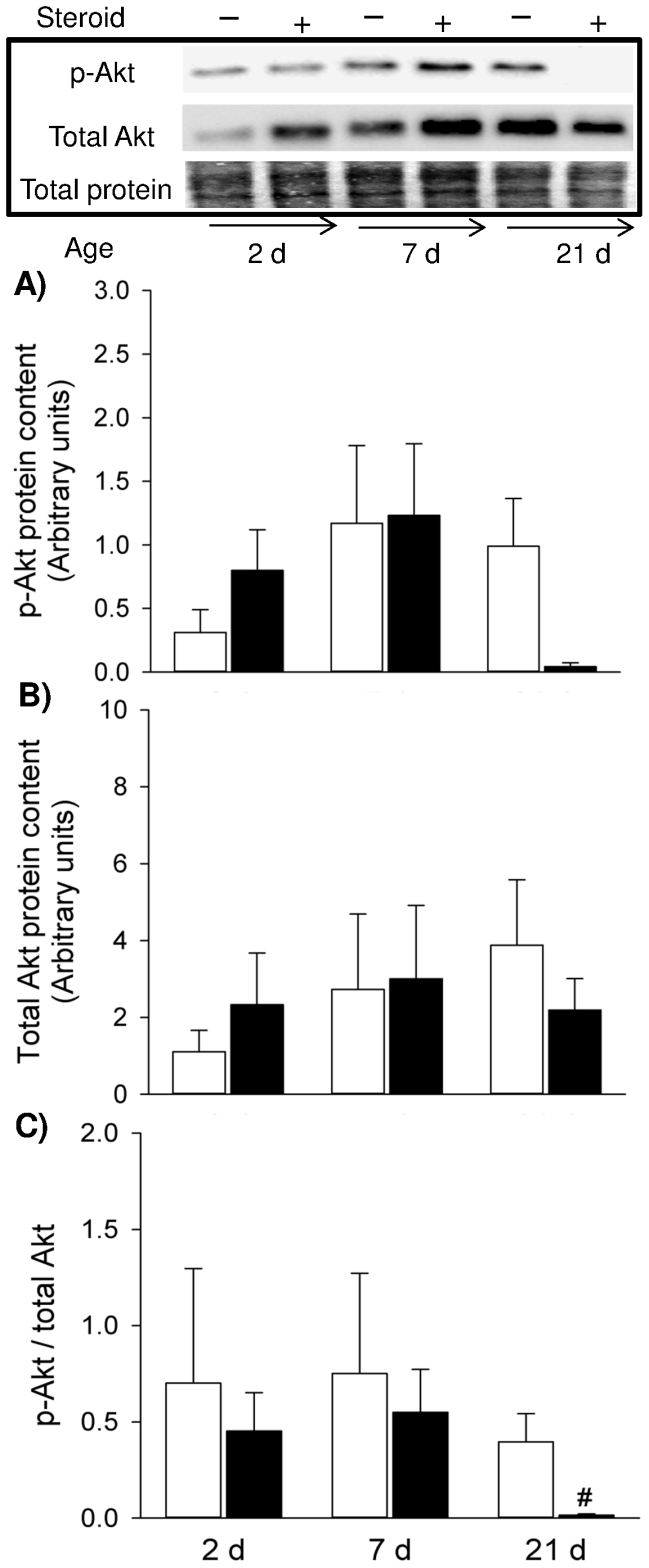
Activity of Akt Signalling. Western blots illustrate expression of signalling molecules using representative samples above the graphs. Graphs show p-Akt (A), total Akt (B) and p-Akt/total Akt ratio (C) in the controls and maternal steroid treatment groups of 2 d (n = 4 for both control and steroid groups), 7 d (n = 4 for both control and steroid groups) and 21 d (n = 5 for both control and steroid groups) postnatal age. Values are Mean (SE). White and black bars refer to the control and steroid treatment groups respectively. ^#^ indicates *p*<0.05 compared with 21 d controls. p: phosphorylated.

There was a wide biological variation in p-Akt and total Akt amongst the experimental groups and overall amounts of these proteins were similar in non-treated controls within postnatal development (Figure 5). Prenatal exposure to steroid did not significantly affect p-Akt and total Akt protein expression throughout development (Figure 5A and 5B). However, the p-Akt/total Akt ratio was significantly reduced after steroid treatment in 21 d pup diaphragm (*p*<0.01) (Figure 5C).

**Figure 6:**
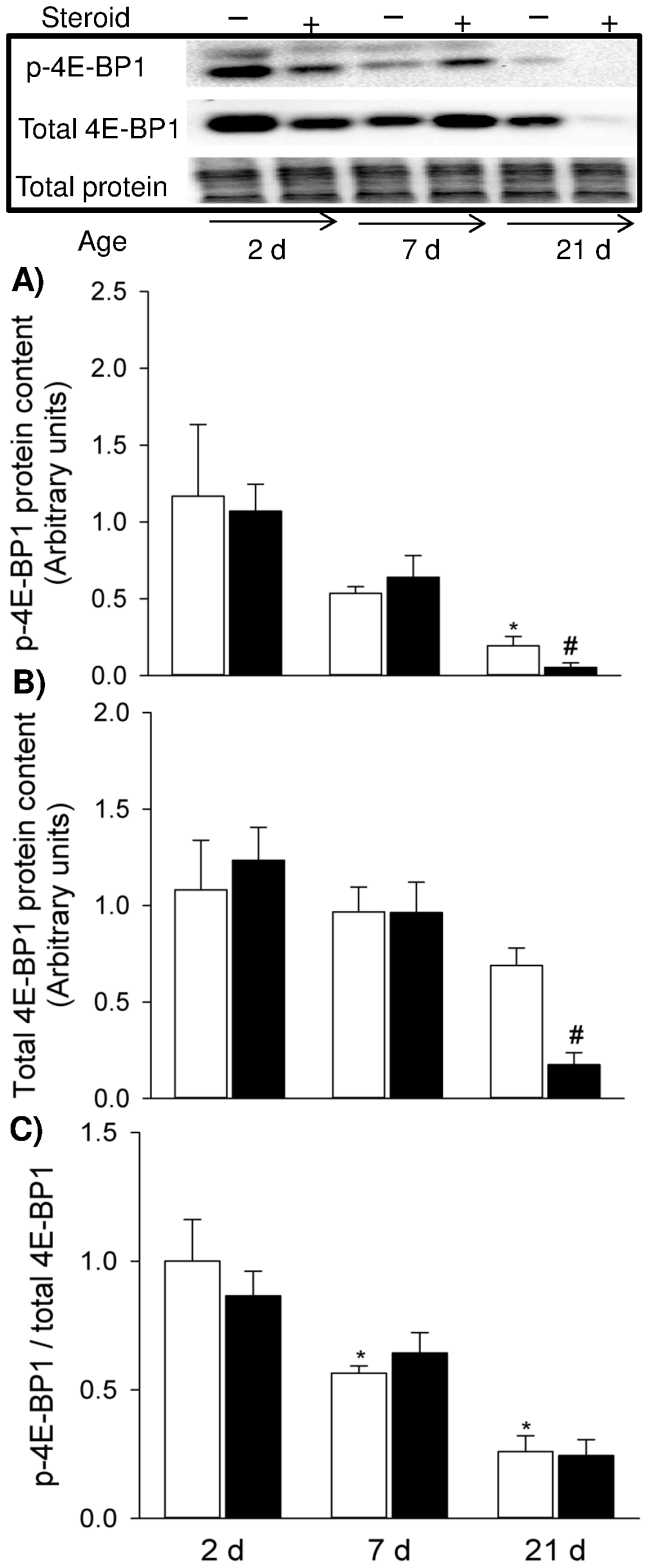
Activity of 4E-BP1 Signalling. Western blots illustrate expression of signalling molecules using representative samples above the graphs. Graphs show p-4E-BP1 (A), total 4E-BP1 (B) and p-4E-BP1/total 4E-BP1 ratio (C) in the controls and maternal steroid treatment groups of 2 d (n = 4 for both control and steroid groups), 7 d (n = 4 for both control and steroid groups) and 21 d (n = 5 for both control and steroid group) postnatal age. Values are Mean (SE). White and black bars refer to the control and steroid treatment groups respectively. ^* #^ indicates *p*<0.05 compared with controls of 2 d and 21 d respectively. p: phosphorylated.

With postnatal growth, there was gradual decrease of p-4E-BP1 and p-4E-BP1/total 4E-BP1 ratio (*p*<0.001) in 2 d to 21 d diaphragm (Figure 6): p-4E-BP1 and the p-4E-BP1/total 4E-BP1 ratio were significantly lower in 21 d compared to 2 d PNA (*p*<0.01) (Figure 6A and 6C). The overall decline was approximately 6.0 fold for p-4E-BP1 and 3.9 fold for p-4E-BP1/total 4E-BP1 ratio. Maternal steroid induced a marked reduction in the active form (phosphorylation) of 4E-BP1 (Figure 6A) and total 4E-BP1 (Figure 6B) in 21 d newborn diaphragm (*p*<0.05), whilst ratio of p-4E-BP1/total 4E-BP1 was similar between the control and treatment groups (Figure 6C).

### Expression of Atrophic Genes

**Figure 7:**
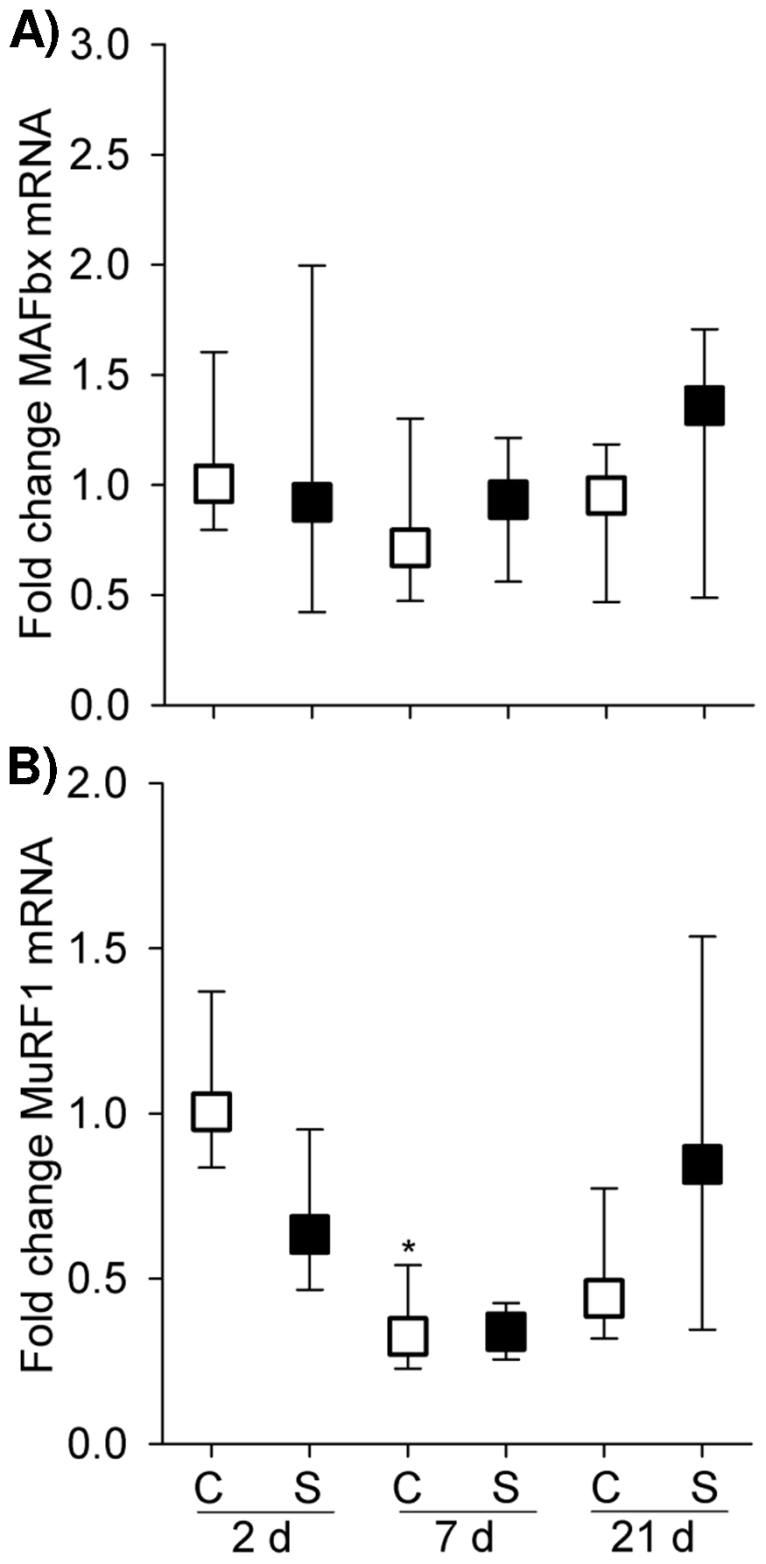
Atrophic Gene Expression. Graphs show MAFbx and MuRF1 mRNA level in the controls and maternal steroid treatment groups of 2(n = 4 for control, n = 8 for steroid group), 7 d (n = 7 for control, n = 4 for steroid group) and 21 d (n = 5 for both control and steroid groups) postnatal age. Values are Median (25^th^, 75^th^ centile), expressed as fold change relative to 2 d control group. White and black box plots refer to the control and steroid treatment groups respectively. ^*^ indicates *p*<0.05 compared with controls of 2 d. C: control; S: steroid.

No significant changes in *MAFbx* gene expression were observed across the different groups (Figure 7A). In contrast, MuRF1 mRNA level was decreased in 7 d naïve diaphragm compared to 2 d control (*p*<0.01) (Figure 7B). Nevertheless, maternal steroid did not affect *MuRF1* gene expression over the postnatal stages examined.

## Discussion

In this study, we show that maternal steroid administration impaired rat pup diaphragmatic contractility and reduced MHC type II muscle fibres at 21 d PNA, but not at 2 d and 7 d PNA. The change of muscle contractile function and fibre type expression is accompanied by depression of the protein synthesis pathway (Akt/4E-BP1). Under control conditions, during 2 d to 21 d postnatal development, we observed a significant MHC fibre transition from MHC neonatal to adult MHC type, particularly increasing fast MHC isoforms and a decreasing trend in key molecules associated with protein synthesis and degradation pathways. Thus we propose that the normal developmental changes that occur in MHC phenotype and protein metabolic signalling during development may exacerbate the effects of antenatal steroid administration on the postnatal diaphragm, which leads to later onset of impaired muscle contractility (21 d).

Our observation of compromised diaphragm contractile function after *in utero* steroid exposure supports our hypothesis that antenatal steroid treatment impairs postnatal respiratory muscle function. In this study, we found no difference in maximal specific force between control and betamethasone treated groups at all neonatal stages studied, but did find marked reductions in submaximal force after betamethasone exposure in the 21 d group. This is a different pattern of effects to that seen in adult rats. However, the effects of glucocorticoid exposure on force production in adults are very variable: decreases in both maximal and submaximal force production [3], decreases in only maximal force production [4] or no effect [5] are reported.

The decrease in submaximal force of 21 d diaphragm in response to betamethasone exposure shown here at stimulation frequencies of 5–20 Hz could be important *in vivo*. In adult rats, the mean peak firing frequency of phrenic motor units measured during normal spontaneous breathing in adult rats is reported to be approximately 26 Hz, with 56% of neurons firing at frequencies below 25 Hz [23]. If diaphragm stimulation frequencies in young (21 d) rats are similar to adult values, force output of diaphragm fibres in betamethasone - exposed 21 d rats, could be 15% (20 Hz) to 40% (5 Hz) lower than control force output *in vivo* (Figure 2A). If so, diaphragm fibres from the betamethasone - exposed rats may require more intense activation to maintain adequate ventilation, making them more prone to fatigue and possible failure.

The reduction in submaximal force seen in the betamethasone treated diaphragm preparations in this study could be due to either changes SR Ca^2+^ handling or decreased sensitivity of the contractile apparatus to Ca^2+^. Glucocorticoid treatment has been reported to alter Ca^2+^ handling in isolated skeletal muscle SR vesicles by some groups [24], but not others [25]. Altered SR Ca^2+^ handling should affect contraction and/or relaxation times. However, we found no difference in normalised TTP and ½ relaxation values between the 21 d betamethasone and control groups, which suggests that SR Ca^2+^ release and reuptake are not significantly affected by betamethasone exposure under the conditions of this study. Therefore, betamethasone could be acting to decrease submaximal force by decreasing the sensitivity of the contractile apparatus to Ca^2+^ in the neonatal diaphragm. In support of this, glucocorticoid exposure decreases the sensitivity of the contractile apparatus to Ca^2+^ adult rat diaphragm muscle fibres, although only in type I fibres [26].

MHC is a critical structural and enzymatic component of the contractile apparatus of muscle [19]. During early postnatal development in rat diaphragm muscle, significant transitions in MHC isoform expression occur that are associated with fibre growth and increased MHC protein content [19]. Expression of MHC isoform mRNA showed that the neonatal MHC isoform gradually decreased while MHC IIb and IIx levels increased and MHC I remained relatively constant throughout postnatal development, which is in good agreement with the developmental pattern of MHC specific isoform transcripts previously reported in the rat [21]. Both MHC I and II protein expression steadily increased with maturation. The apparent inconsistency in MHC I mRNA and protein expression during development is not unexpected as it was proposed that MHC protein expression was not solely under transcriptional control in the developing rat diaphragm except for the MHC neonatal isoform [21].

From birth to the first week PNA, muscle fibres are predominantly composed of neonatal MHC [19]. Analogous to preterm humans, the neonatal rat diaphragm (21 d old) has a high proportion of fast type II fibres [16], with only 10%–20% type I fibres [16], [19], while neonatal MHC almost disappears during this period. The developmental change of muscle fibres explains the increase in submaximal force at 21 d during development (Figure 1A) and susceptibility of 21 d diaphragm to maternal steroid injury. MHC IIb and MHC IIx expression is positively correlated with muscle force generation [27], [28]. We observed a dramatic increase in both MHC IIb and IIx isoform mRNA over development, particularly compared to 2 d diaphragm. Consequently the enhanced submaximal strength at 21 d PNA may be due in part to the emerging and predominant expression of MHC IIb and MHC IIx isoforms.

Glucocorticoids cause selective atrophy of fast-twitch or type II muscle fibres (particularly IIb and IIx) with less or no impact observed in type I fibres [3], [6]–[8]. Therefore, the glucocorticoid detrimental effect is more pronounced in the diaphragm muscle (predominantly fast twitch fibres) than the soleus muscle (predominantly slow twitch muscle) [8]. Coincident with the functional change, expression of MHC IIb mRNA and type II fibre protein was markedly reduced in 21 d diaphragm following antenatal glucocorticoid exposure. Given the positive correlation between MHC II protein and contractile function [27], [28], the loss of MHC II could explain, at least in part, the functional deficit that was present in 21 d pups after antenatal exposure to maternal glucocorticoid injections. It is likely that there is a significant reduction in total MHC protein in the steroid-exposed muscles as MHC I also showed a decreasing trend. Altered conformation in MHC composition or reduced absolute number of contractile proteins may affect cross-bridge cycling kinetics, the force generated per cross bridge, or even myofibrillar density and the energetic demands of the muscle contractile proteins, subsequently influencing functional properties such as specific force generation, maximum unloaded velocity of shortening and fatigue resistance [19].

To explore further how muscle fibre protein loss is regulated in response to maternal steroid during muscle development, we selectively observed key components in the protein synthesis pathway (Akt and 4E-BP1) and the proteolytic markers MAFbx and MuRF1 (UPP E3 ligase). In 21 d pup diaphragm, maternal betamethasone exposure reduced the phosphorylation of Akt and 4E-BP1, suggesting that such insult impaired protein synthesis pathway which explains the reduced muscle contractility and muscle fibre loss that occurred at the same time point. As gene expression of MAFbx and MuRF1 was not altered, UPP as a principal protein degradation pathway seemed to be unaffected responding to the steroid insult. Additionally, autophagy also is a significant contributor to protein degradation. Further study is needed to determine if protein degradation is activated via an autophagy mechanism.

During muscle protein synthesis, phosphorylated 4E-BP1 promotes elf4E release from the inactive elF4E·4E-BP1 complex, allowing the formation of the active elF4G·elF4E complex, which mediates the binding of mRNA to the 43S ribosomal complex [29]. Thus, decreased activation of 4E-BP1 induced by maternal betamethasone exposure could suppress protein synthesis rates which are dependent upon the ribosomes number in the tissue and efficiency with which the ribosomes translate mRNA into protein [30]. The corresponding change observed in Akt signalling implies that Akt was an upstream regulator in modulating 4E-BP1 activity during antenatal steroid induced diaphragm dysfunction, probably through the classical Akt/mTOR pathway.

In the developing muscle, protein synthesis is the primary regulator of protein accretion [31]. Even minor alterations in protein synthesis rates could substantially reduce muscle protein accretion [32]. Both anabolic (4E-BP1) and catabolic (MuRF1) signalling showed high baseline activity during early PNA (2 d) and decreased as muscle maturation advanced. These findings were consistent with our previous observation that protein synthesis and degradation signalling experienced a parallel change within development [13]. However, the high growth rate of neonatal muscle is attributable to the high rate of protein synthesis owing to dominant rates of protein synthesis over degradation [31]. Orellana et al. [33] revealed that in the early postnatal stage skeletal muscle maintains a high anabolic drive and catabolic signal activation that cannot be disrupted by endotoxin, but as maturation advances, the effect of endotoxin on muscle protein catabolism manifests its severity. In the current study, we consistently observed functional and phenotypic injury and attenuation of anabolic signalling at PNA 21 d, but not in 2 d and 7 d. The absence of a response to maternal steroid in 2 d and 7 d PNA may be also related to high baseline activity of protein metabolic signalling which confers protection to newborn diaphragm at early postnatal period, whilst the postnatal development aggravates impairment of protein synthesis and manifestation of muscle injury. Of note, Akt signalling did not seem to play a significant role in postnatal muscle development as both phosphorylated Akt and total Akt remained unchanged from 2 d to 21 d PNA. Apart from driving protein synthesis through activating mTOR and its downstream effectors (p70S6K and 4E-BP1), Akt impedes protein degradation by inhibiting atrophic gene expression. However, within normal postnatal development down-regulation of 4E-BP1 signalling and *MuRF1* gene expression observed in the present study is not likely to be modulated under Akt signalling. Therefore the alternative mechanisms may exist, e.g. Leucine–stimulated signal to activate 4E-BP1 pathway [34], regulation of atrophic gene via reactive oxygen species activated FOXO and NF-κB [35].

In summary, the results of this study indicate that antenatal betamethasone exposure results in significant remodelling of the diaphragm by postnatal day 21, at the same time normal developmentally-mediated changes in the tissue are occurring. Normal developmental increases in the level of protein synthesis were attenuated by betamethasone exposure, resulting in decreased levels of MHC II and presumably other contractile proteins [36], which results in inhibition of diaphragm force output. The deleterious effect of maternal steroid treatment on postnatal pup diaphragm indicates that the clinical use of betamethasone may contribute to respiratory insufficiency in the preterm infant by inducing respiratory muscle dysfunction.
